# *in vivo* cellular evidence of autophagic associated spermiophagy within the principal cells during sperm storage in epididymis of the turtle

**DOI:** 10.18632/aging.103144

**Published:** 2020-05-15

**Authors:** Imran Tarique, Yonghong Shi, Noor Samad Gandahi, Baitao Ding, Ping Yang, Chang Chen, Waseem Ali Vistro, Quisheng Chen

**Affiliations:** 1 MOE Joint International Research Laboratory of Animal Health and Food Safety, College of Veterinary Medicine, Nanjing Agricultural University, Nanjing 210095, Jiangsu Province, China; 2 Shanghai Veterinary Research Institute, Chinese Academy of Agricultural Sciences, Shanghai 201203, China

**Keywords:** principal cells, spermiophagy, autophagy, epididymis, turtle

## Abstract

The epididymis plays a significant role as a quality control organ for long-term sperm storage, maturation, and fertilizing ability and perform filtration function to eliminate abnormal or residual spermatozoa by phagocytosis. However, the role of autophagy in spermiophagy during sperm storage in turtle epididymis still needs to be studied. In this study, we reported *in vivo* spermiophagy via the cellular evidence of lysosome engulfment and autophagy within the principal cells during sperm storage in the turtle epididymis. Using immunofluorescence, Lysosome associated membrane protein-1 (LAMP1) and microtubule-associate protein light chain 3 (LC3) showed strong immunosignals within the apical cytoplasm of epididymal epithelia during hibernation than non-hibernation. Co-immunolabeling of LAMP1 and LC3 was strong around the phagocytosed spermatozoa in the epididymal epithelia and protein signaling of LAMP1 and LC3 was confirmed by western blotting. During hibernation, ultrastructure showed epididymal principal cells were involved in spermiophagy and characterized by the membrane’s concentric layers around phagocytosed segments of spermatozoa, degenerative changes in the sperm head and lysosome direct attachment, and with the existence of cellular components related to autophagy (autophagosome, autolysosome). In conclusion, spermiophagy occurs by lysosomal engulfment and autophagic activity within the principal cells of the turtle epididymis during sperm storage.

## INTRODUCTION

Generally, spermatozoa that develop within testicular seminiferous tubules are morphologically complete when released, but immotile and unable to fertilize an oocyte [1]. After spermiation, the spermatozoa travel via efferentes ductuli into the epididymis for post testicular maturation where they develop the capacity for motility and acquire fertilizing ability [2]. The epididymis epithelial such as principal cell, basal cell, apical cell, narrow cell, and halo cells creates a unique microenvironment for the spermatozoa to gain fertilizing ability [2, 3], and in many species the mature spermatozoa are stored within the cauda epididymis / vas deferens until they are ejaculated. Except in human, there is scant data about spermatozoa that can achieve the ability to fertilize without passing through epididymis segments [4]. The phenomenon of sperm maturation in the epididymis has been well documented in mammals [5–7] and it has been suggested that the epididymis functions as a quality-control organ to prevent the inclusion of misshapen, genetically abnormal or infertile spermatozoa in the ejaculate [4]. In the epididymis of mammals, weak and defective sperms incorporated within the cytoplasm of epithelia, where epithelial cells released ubiquitin that binds with defective spermatozoa for phagocytosis process [8, 9]. In most vertebrates, prior to or during mating, not all spermatozoa are capable of being ejaculated from the reproductive tract due to their weak capability of energetic disorder of mitochondria or DNA damage [10] or immaturity, and these are referred as residual or unejaculated spermatozoa. Such spermatozoa are eliminated by the duct epithelia or luminal macrophages in the male reproductive tract via phagocytosis or absorbing [4]. Phagocytosis of the spermatozoa has been regarded as a deliberate and active process of consumption of entire spermatozoa or their fragments [11]. This activity has been reported to occur in various groups of vertebrates such as reptiles [11, 12], birds [13, 14], amphibians [15], teleosts [16] and mammals [17–21]. While in the turtle epididymis stored spermatozoa appear to deteriorate with the passage of time, while *in vitro* stored epididymal spermatozoa at 4°C for 40 days showed oxidative damage to mitochondria and results in apoptotic like changes [22, 23]. Therefore, we hypothesize that during sperm storage in the epididymis of soft-shelled turtle (*Pelodiscus sinensis*), epithelial cells also play their potential role to eliminate spermatozoa via phagocytosis.

Phagocytosis has been defined as the cellular uptake of particles by endocytosis [24]. Together this, uptake of exogenous substances has features in common autophagy, and endogenous processes of sequestration and lysosomal disposal [25]. Collectively, both processes involve lysosomal degradation but with different structural cellular appearance, such as i) phagophores characterized as lipid bilayer in autophagy but in phagocytosis it is a single membrane structure, ii) the digesting vesicles in autophagy are referred to as autolysosome, and phagolysosomes in phagocytosis [26]. To study phagocytosis, Lysosomal Associated Membrane Protein-I (LAMP1) and Microtubule-associated protein light chain-3 has been suggested as the essential protein marker for lysosome fusion with phagosomes and autophagosome respectively [27, 28]. Recently, LC3-associated phagocytosis has enabled us to glimpse features of immune regulation and inflammatory responses across various cells and tissue types [26, 29]. In the epididymis, the role of autophagy and phagocytosis still needs much attention to evaluate the interaction between spermatozoa and epididymal epithelium. However, the role of autophagy in the male reproduction system is currently under great attention and suggested for broad range of the cellular events such as in spermatogenesis, degradation of sperm cytoplasmic contents and testosterone biosynthesis [30].

In ectothermic animals, spermatozoa are produced at much slower rates, therefore such animals are very reliant on establishing a store of spermatozoa for use during the mating season. Unlike mammals, in many reptiles such as snake [31] and turtle [23] have evident sperm storage in the epididymis. The reproductive activity in *Pelodiscus sinensis* turtle is seasonal, spermatogenesis starts during late May and end with spermiation in October. Immature spermatozoa are transferred into the epididymis, where they are stored until the next mating season [32]. Previous studies by our research group have shown that spermatozoa are stored in the epididymis, and they interact with the epididymal epithelia of *P. sinensis*, [3, 32]. Recently Chen et al., concluded that lipophagy contributes to lipid droplet breakdown for long term sperm storage in the epididymis of *P.sinensis* [33]. Collectively, the data on residual or unejaculated spermatozoa phagocytosed by the epididymal epithelia in the *P.sinensis* need to be studied and also to explore the role of autophagy in eliminating of endocytosed spermatozoa. This perspective will help to understand the autophagic phagocytosis of spermatozoa in the epididymis during long-term storage. Therefore, present study analyzed spermiophagy at light and ultrastructural levels with western blotting to evaluate the phagocytosis of spermatozoa by lysosome degradation and autophagy within the epididymal epithelia of *P.sinensis.*

## RESULTS

### Light and Fluorescent microscopy of the epididymis

**Figure 1:**
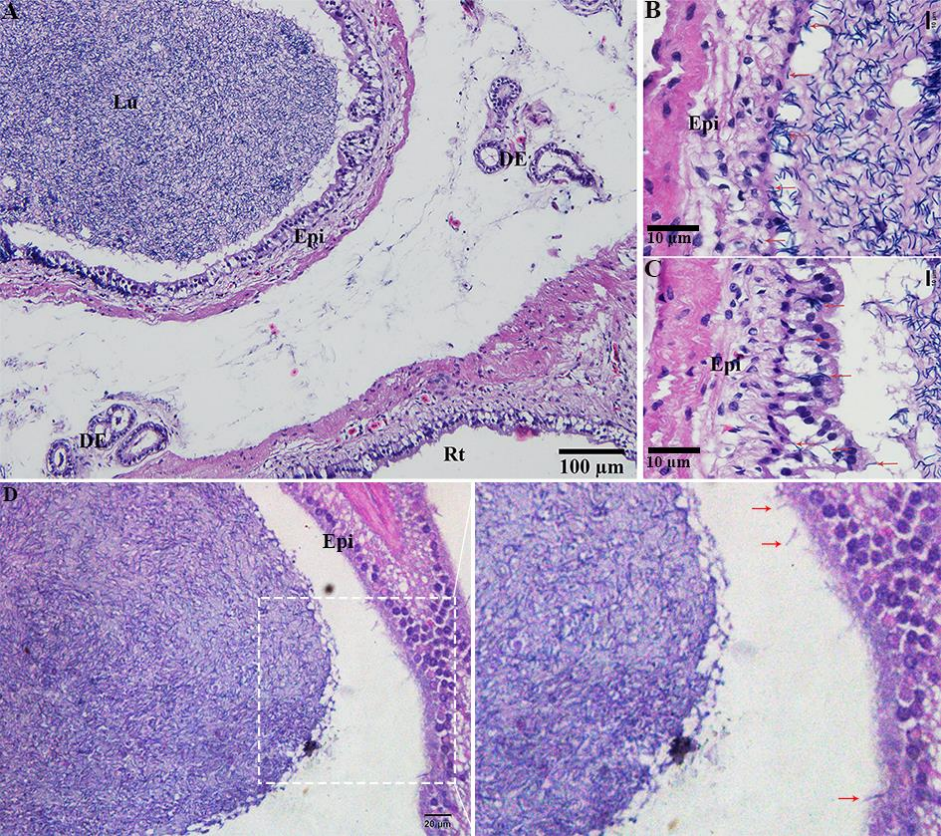
**Light microscopy of epididymis within the testis of turtle.** Spermatozoa storage during hibernation (**A**–**C**) and non-hibernation. (**D**) Red arrow showed interaction of spermatozoa with epididymal epithelia. DE: ductuli efferent; Epi: Epididymis; Lu: Lumen; Rt: rete testis. Scale bar: (**A**) 100 μm, (**B**, **C**) 10μm, (**D**) 20 μm.

**Figure 2:**
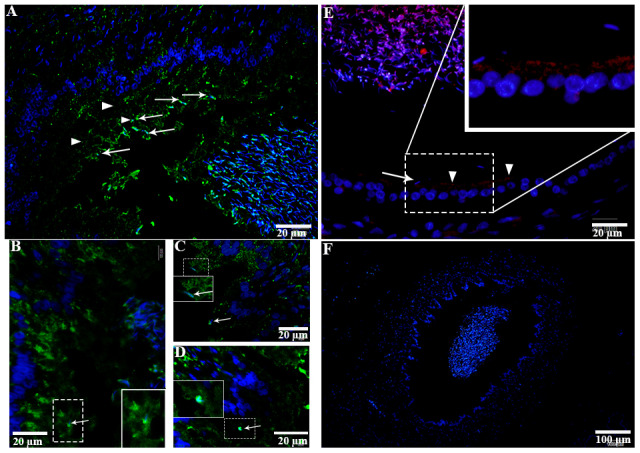
**LAMP1 localization in the epididymis of turtle.** LAMP1 immunolabeling (white arrowhead) in the epididymis during hibernation (**A**–**D**) and non-hibernation (**E**) PBS served as negative control. (**F**) White arrow indicates interaction of luminal spermatozoa with epithelia of epididymis. Rectangular area showed enlarged area. Scale bar: (**A**, **B**, **E**) 20 μm and (**C**, **D**) 10 μm.

**Figure 3:**
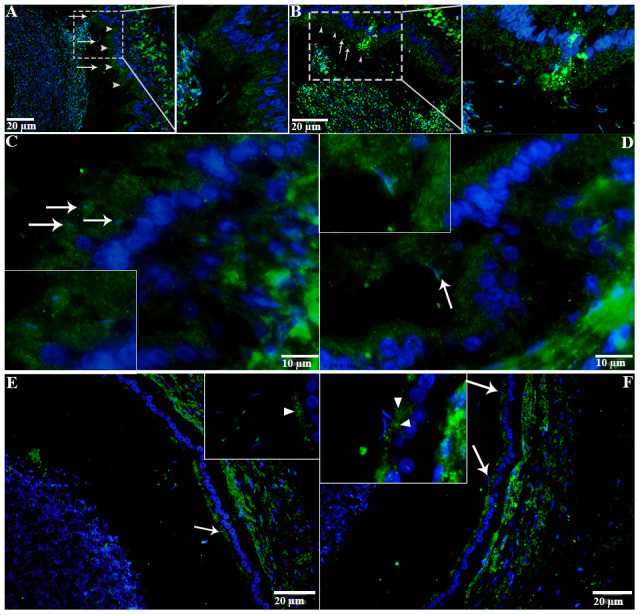
**LC3 localization in the epididymis of turtle.** LC3 immunolabeling (white arrowhead) in the epididymis during hibernation (**A**–**C**) and non-hibernation. (**E**, **F**) White arrow indicates interaction of luminal spermatozoa with epithelia of epididymis. Rectangular area showed enlarged area. Scale bar: (**A**, **B**, **E**, **F**) 20 μm and (**C**, **D**) 10 μm.

**Figure 4:**
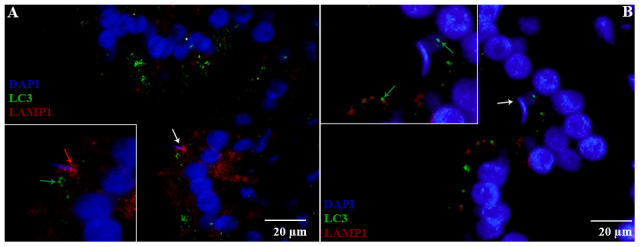
**Double immunofluorescence of LC3 and LAMP1 in the epididymis of turtle.** Immunolabeling of LC3 (green arrow) and LAMP1 (red arrow) during hibernation (**A**) and non-hibernation. (**B**) White arrow indicates spermatozoa interaction with apices of epididymal epithelia. Scale bar: (**A**, **B**) 20 μm.

**Figure 5:**
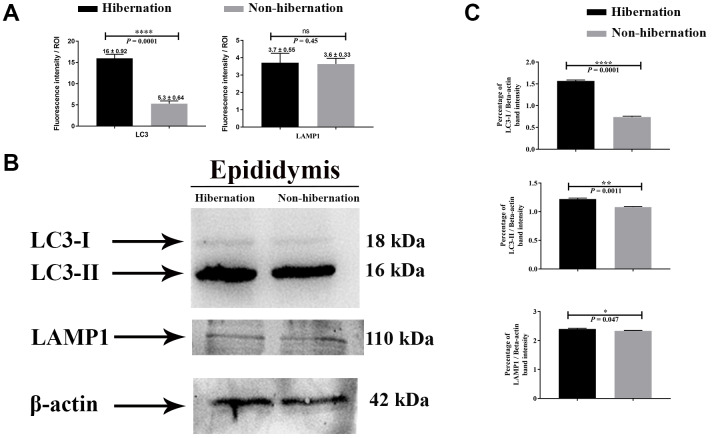
**Protein immunostaining in the epididymis of turtle.** Fluorescent intensity (**A**) and immunoblots protein expression (**B**, **C**) of LC3 and LAMP1 in epididymis during hibernation and non-hibernation period. Data presented as mean ± S.E.M. NS: non-significant.

H&E staining revealed the transit passage of spermatozoa, that develop at the seminiferous tubule, travel via the rete testis to the ductuli efferentes and are stored in the lumen of the epididymis during hibernation (Figure 1A–1C) and non-hibernation (Figure 1D). Higher magnification showed that numerous spermatozoa were in-contact with the epididymal epithelia (Figure 1B), whereas few spermatozoa were observed within the cytoplasm of the epididymal epithelia (Figure 1C). These spermatozoa were believed to be residual or abnormal as they were phagocytosed by the epididymal epithelia. Whereas during non-hibernation, we observed similar interaction of spermatozoa with apices of epididymal epithelial (Figure 1D). To investigate the spermiophagy within the epididymal epithelia of the turtle, we used immunofluorescence to determine the expression of LAMP1, which is a key lysosomal marker. It was observed that, LAMP1 strongly localized within the supranuclear cytoplasm of the epididymal epithelia and in the luminal spermatozoa during the hibernation (Figure 2A). Detailed observations during hibernation suggest that, LAMP1 is strongly expressed around the spermatozoa which is observed within the apical epididymal epithelium (Figure 2B–2D). While during non-hibernation, the immunolabeling of LAMP1 was moderate at the apices of epididymal epithelia (Figure 2E). While PBS was served as negative control (Figure 2F). Together with this, we also investigated autophagy by using immunofluorescence to determine the LC3 expression within the epididymal epithelia. Results showed that, strong LC3 immunolabelling in the luminal spermatozoa and within the apical cytoplasm of epididymal epithelia during the hibernation. Whereas during non-hibernation, the immunopositivity of LC3 was weak in luminal spermatozoa and in the apical cytoplasm of epididymal epithelia (Figure 3A, 3B). Immunolabeling of LC3 was also observed around the spermatozoa within the apical cytoplasm of epididymal epithelia during the hibernation (Figure 3C, 3D). Whereas during non-hibernation, weak immunopositivity of LC3 observed in the luminal spermatozoa and in the apices of epididymal epithelia (Figure 3E, 3F). Moreover, we performed double immunolabeling of LC3 and LAMP1 to target the phagocytosis of spermatozoa by epididymal epithelia. At apices of epididymal epithelia, we observed spermatozoa with strong labeling of LAMP1 and LC3 during hibernation (Figure 4A), while modestly during non-hibernation (Figure 4B) in the epididymis of turtle. Furthermore, quantification fluorescent intensity of LC3 and LAMP1 were significantly observed (Figure 5A). The immunoblots protein expression was performed and confirmed the protein signaling of LAMP1 and LC3A/B (Figure 5B, 5C), which demonstrate higher band densities during hibernation than non-hibernation in the epididymis. Overall, results suggest that, strong immunopositivity of LAMP1 and LC3 occurs within the supranuclear cytoplasm and around the spermatozoa which is located within the epididymal epithelium during the hibernation than non-hibernation period of turtles. These results suggest that epididymal epithelial cells are involved in spermiophagy during sperm storage in the turtle.

### Ultrastructure of the epididymis and spermatozoa during hibernation

**Figure 6:**
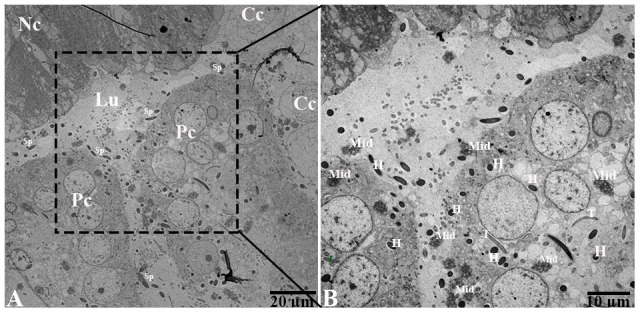
**Transmission electron micrograph of epididymis during hibernation.** Cc: clear cell; Lu: lumen, Nc: narrow cell, Pc: principal cell, Sp: spermatozoa (H: head, Mid: midpiece, T: tail). Scale bar: (**A**) 20 μm and (**B**) 10 μm.

**Figure 7:**
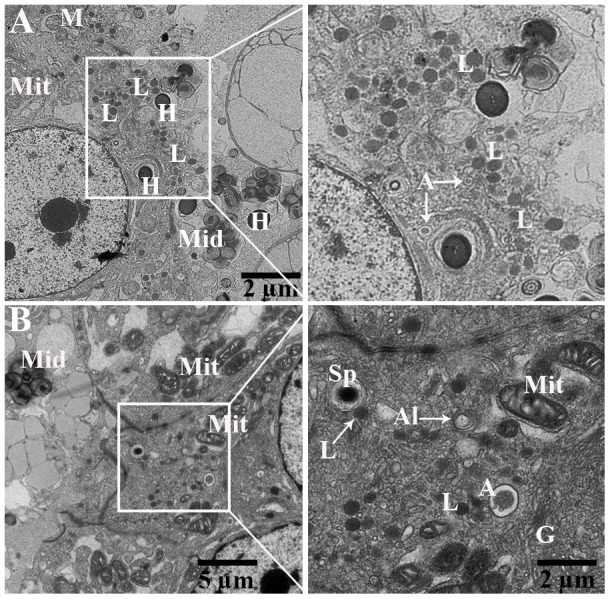
**Transmission electron micrograph of Spermatozoa within cytoplasm of principal cells of epididymis.** Al: autolysosome; G: Golgi complex; L: lysosome, Mit: mitochondria, Sp: spermatozoa (H: head, Mid: midpiece, T: tail). Rectangular area showed enlarged area. Scale bar: (**A**) 5 μm and (**B**) 5 μm and 2 μm.

Detail ultrastructural study was performed during hibernation to characterize the autophagic phagocytosis in the epididymal epithelia. TEM results showed that the epididymis consists of various cells such as principal cells, clear cells, and narrow cells, and numerous spermatozoa were in the lumen of the turtle epididymis. Detailed TEM observations (Figure 6) indicated that various sperm segments (head, midpiece and tail) were scattered within the cytoplasm of principal cells while in the lumen, some spermatozoa were associated with the apical border of principal cells (Figure 6A, 6B). Furthermore, within the apical cytoplasm of principal cells, numerous lysosomes, mitochondria, Golgi complex, vacuolization, and segments of phagocytosed spermatozoa were seen. Lysosomal and autophagic activity were also readily observed (Figure 7A, 7B).

### Phagocytosis of the spermatozoa within the principal cells of the epididymis during hibernation

**Figure 8:**
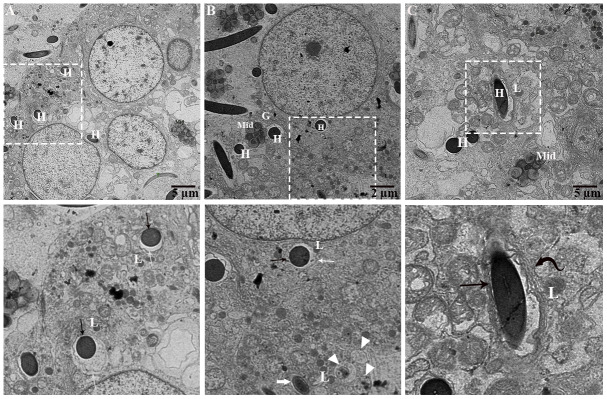
**Ultrastructure: Formation of concentric layer around sperm heads undergoing engulfment.** Al: autolysosome; Cd: cytoplasmic droplet of spermatozoa, L: lysosome, Mit: mitochondria, Sp: spermatozoa (H: head, Mid: midpiece, T: tail). Rectangular area showed enlarged area. Black arrow: interconnected zone, White arrow: electron translucent zone, Curved black arrow: numerous concentric layers around spermatozoa head, Thick white arrow: reduced electron translucent zone, Arrowhead: autophagic activity. Scale bar: (**A**, **C**) 5 μm and (**B**) 2 μm.

**Figure 9:**
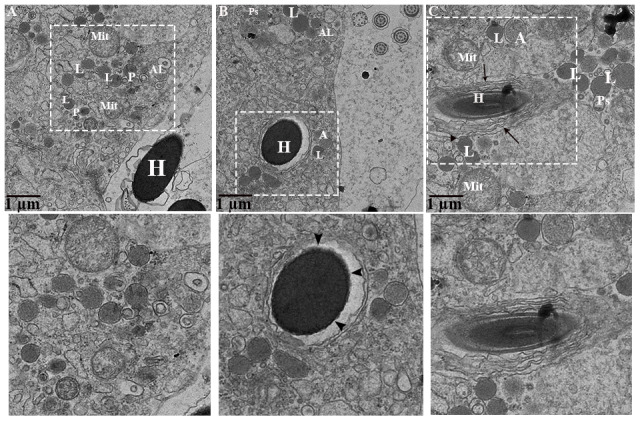
**Ultrastructure: Lysosomal and autophagic activity around spermatozoa.** A: autophagosome; Al: autolysosome; G: Golgi complex; H: Head of spermatozoa; L: lysosome, Mit: mitochondria; P: phagolysosome. Rectangular area showed enlarged area. Arrow: numerous concentric layers around spermatozoa, Sharp arrow: degenerate segment of spermatozoa, Arrowhead: lysosomal attachment with spermatozoa. Scale bar: (**A**–**C**) 1 μm.

During phagocytosing, segments of spermatozoa have become contained within vacuoles that are separated from the principal cell cytoplasm by an electron lucent space. On the other side, the principal cell cytoplasm exhibited no distinct space but direct attachment with the plasma membrane of the vacuolated segment of spermatozoa (Figure 8A). These two zones represented as two concentric layers around the sperm segment inside the principal cytoplasm (Figure 8A–8C). Interestingly, lysosomal activity (lysosomes, engulfment by lysosome and phagolysosome) and autophagy (autophagosome and autolysosome) can also be observed (Figure 9A). It was observed that the degradation occurred in the head segment of phagocytosed spermatozoa, which is no more resistant to the degradation as shown in the Figure 9B, 9C, the disruption in the nuclear plasma membrane. Under TEM, it is characterized by the formation of membranous concentric layers around the head segment and acrosomal sheath. As phagocytosis processes, the concentric layer disappears and finally there is direct contact between principal cytoplasm and the spermatozoa. Moreover, we also observed direct attachment of the lysosome and the concentric layers around the phagocytosed segment of spermatozoa and existence of autophagy component (autophagosome and autolysosome) (Figure 9). Ultrastructure results suggest that spermiophagy is active during sperm storage within the principal cells of epididymis. Spermiophagy is characterized by membranes concentric layers around the phagocytosed segment of spermatozoa and direct attachment of lysosome for engulfment with the autophagic activity.

## DISCUSSION

The phenomenon of sperm maturation within the epididymis and preventing misshapen, genetically abnormal or infertile spermatozoa from entering the ejaculate, has inferred that this organ has a role in quality control [4–7]. However, lysosomal engulfment has been reported in different segments of the male and female reproductive tract. Autophagy, which is currently under great debate and suggested in elimination of dying cells and pathogens [29]. More importantly, lysosome and autophagy engage in a unique endogenous process of sequestration and lysosomal disposal [25]. Nevertheless, there is no study exploring the lysosomal and autophagic elimination of spermatozoa within the epididymal epithelia of the turtle. In the current study, we characterized *in vivo* spermiophagy by lysosomes and autophagy within the principal cells of the turtle epididymis. We used specific markers for lysosomes (LAMP1) [27] and autophagy (LC3) [28] to confirm the lysosomal and autophagic activity through immunofluorescence and western blotting. Furthermore, TEM was used for the ultrastructural observation of phagocytosed spermatozoa by lysosome and autophagy within the principal cell of the turtle epididymis.

In the Chinese soft-shelled turtle, the epididymal epithelium consists of principal, narrow, apical, clear, and basal cells; among them principal and clear cells are especially abundant in the lining of the epididymis. Principal cells are known for absorption and secretion in all cranial, middle and caudal segments of the epididymis [3]. Unlike mammals and birds, turtle spermatozoa are known to be stored in the epididymis for long periods (6-7 months) [34]. In the current study, the sampling was done from hibernation (Nov-May) and non-hibernation (Aug-Sept), hence the results of this study showed spermatozoa stored within the lumen of corpus epididymis. Gist et al., concluded that turtle (*Chrysemys picta*) epididymis is a primarily storage organ and spermatozoa present throughout the year [23]. In reptiles, the cauda epididymis of *S. ponticeriana* has been suggested as spermiophagic [12], and vesicular cells of *Chrysemys picta* were seen to be involved in spermiophagy [35]. Together with this, our results demonstrate that principal cell of the middle part of epididymis was active in spermiophagy, which might be due to these cell’s absorptive function [3]. Therefore, it can be suggested that principal cells also have spermiophagic capability.

The acrosome is a specialized lysosome-related organelle located over the anterior part of the sperm nucleus that is highly conserved throughout evolution [36, 37]. In the middle piece of human ejaculated spermatozoa [37] and turtle epididymal spermatozoa [33], the autophagy-related protein such as LC3 was involved in mobility, motility and lipid droplet utilization for longevity. Results of present study showed LAMP1 and LC3 markers positively immunolabelled the spermatozoa in the lumen of the turtle epididymis. The highest activity of lysosomal enzymes has been reported in the middle segment of the mammalian epididymis (ram, rabbit, rat and hamster) within the supranuclear cytoplasm of the epididymal epithelium [38]. The role of LAMP1 has been suggested as mandatory for the fusion of lysosome with a phagosome [27], therefore playing an important role in the lysosome mediated physiological processes. During autophagy, cytosolic LC3 (LC3-I) is modified to its membrane bound form (LC3-II) located on the autophagosome [28]. Although LC3 immunolabeling has been studied in various epithelia. In the current study, our results showed LAMP1 and LC3 immunolabeling within the supranuclear cytoplasm of the middle part of the epididymal epithelium of the turtle. More interestingly, strong immunolabeling of these markers around the phagocytosed spermatozoa within the cytoplasm of epididymal epithelia were observed. Though we speculate that these spermatozoa were abnormal or residual that were subjected for elimination by lysosomal and autophagy activity.

Moreover, the present study provides ultrastructural findings of the phagocytosed spermatozoa by the principal cell of the turtle epididymis. Subsequently, membranes concentric layers around, and degenerated changes in the head piece of phagocytosed spermatozoa. While lysosomal direct attachment and existence of autophagy components (autophagosome, autolysosome) with the phagocytosed spermatozoa within the principal cell of the turtle epididymis. All these ultrastructure findings with immunolabeling of LAMP1 and LC3, suggest that lysosome actively participates in phagocytosis process of weak or immotile spermatozoa and autophagy play a significant role in assisting this process. In previous studies, spermiophagy was proposed based on ultrastructural findings in the male and female reproductive tracts of animal groups such as birds [13, 39], amphibians [15], reptiles [11, 12, 35], teleosts [16] and mammals [17–21]. In the epididymis of sub-fertile hybrid mice, principal cells were shown to be involved in the phagocytosis of mature and immature spermatozoa [21]. In the rat epididymis the aging effect was observed at cellular level and suggested that principal cells exhibited with more lysosomes and large vacuoles [40]. Whereas *in-vitro* epididymal spermatozoa from turtle showed apoptotic like changes when stored at 4°C for 40 days [22]. Furthermore, numerous lysosomes, mitochondria, Golgi complex, vacuolization form a unique environment around the endocytosed segments of spermatozoa. It is suggested that, hydrolytic enzymes of lysosome processed in Golgi complex to form primary lysosome that attached with selective endocytosed vacuole or endocytosed vacuole fused by the secondary phagolysosome that generated from Golgi complex [41].

In conclusion, this study provides the first cellular information about *in vivo* spermiophagy by lysosome and autophagy within the principal cell of the turtle epididymis and presents ultrastructural findings of phagocytosed spermatozoa within the cytoplasm of principal cells, cellular components of lysosome and autophagy. Moreover, we have shown that, lysosome (LAMP1) and autophagy (LC3) specific markers labeling also provide evidence that lysosome and autophagy are both involved in spermiophagy within the principal cells of the turtle epididymis.

## MATERIALS AND METHODS

### Animals

All procedure was conducted in accordance and guideline of Animal Research Institute Committee of Nanjing Agricultural University.

Adult male of Chinese soft-shelled turtle (*Pelodiscus sinensis*) (4-5 years old) were obtained from ponds in Nanjing, China. A total *n=*10 (each group *n* = 5) turtles collected during the hibernation period (Nov to May) and non-hibernation (August-September) were used in this study. The turtles were not fed prior to sample collection. The turtles were anaesthetized with an intraperitoneal injection of pentobarbital sodium (20 mg kg^-1^) and killed by exsanguination. All efforts were made to minimize animal suffering. The study etiquette was approved by the Science and Technology Agency of Jiangsu Province (SYXK (SU) 2010-0005).

### Light microscopy

Sample from corpus (middle) segment of the epididymis were placed in 10% neutral buffered formalin for fixation overnight, and then embedded in paraffin wax. Sectioning was done at 5μm. These sections were stained with hematoxylin and eosin procedures (Harris’s hematoxylin (H & E) for 2 min and 1% eosin for 30 sec) for light microscopy analysis using an Olympus microscope (BX53), camera (Olympus DP73), Japan.

### Fluorescent microscopy

Slides containing 5 μm tissue sections were incubated with rabbit primary antibody (LC3 (12741- Cell Signaling Technology, Danvers, Massachusetts, USA) (1:100 dilution) and LAMP1 (Proteintech (55273-1-AP)) (1:100 dilution). Whereas for negative control phosphate buffer saline (pH 7.2) ((P7059) Sigma-Aldrich, Darmstadt, Germany) was used. Following primary antibody applications, all the samples were incubated with a secondary antibody for 1 hour at 4°C and were rehydrated in phosphate buffer saline (PBS) ((P7059) Sigma-Aldrich, Darmstadt, Germany). The sections were incubated with DAPI (4′,6-diamidino-2-phenylindole = nuclear staining) (catalog no. 13G04A76; Boster, Wuhan, China) and were stimulated under a fluorescent microscope over time. All the specimens were initially viewed by using an LED to visualize fluorescence under the microscope.

### Immunofluorescence double labeling

Slides containing 5 μm tissue sections were deparaffinized followed by antigen unmasking in sodium citrate buffer. Then, the tissue sections were incubated with 1% bovine serum albumin (room temperature for 30 min) to block non-specific antibody binding. Following this, the samples were incubated at 4°C overnight with the following primary antibodies: rabbit anti-LC3 (1:100 dilution) (12741-Cell Signaling Technology, Danvers, Massachusetts, USA) and mouse anti-LAMP1 (1:100 dilution) (15665- Cell Signaling Technology, Danvers, Massachusetts, USA). Then, after washing with 0.1M PBS (pH 7.4), samples were incubated with Alexa Fluor-488-conjugated goat anti-rabbit IgG (1:100 dilution; catalog no. RBaf48801; Fcmacs, Nanjing, China) and Tritc-conjugated goat anti-mouse IgG (1:100 dilution; catalog no. BA1089; Boster, Wuhan, China), secondary antibodies for two hours at 37°C. Nuclei were counterstained with 4′,6-diamidino-2- phenylindole (DAPI) (catalog no. 13G04A76; Boster, Wuhan, China). Visualization of fluorescence on sections were observed under an Olympus BX53 microscope and fluorescent images were captured with an Olympus DP73 digital color camera

### Western blotting

Samples were homogenized in ice-cold with radioimmunoprecipitation assay (RIPA) buffer. The homogenates were subsequently centrifuged at 15,000 g for 10 min at 4°C. Then, the samples were subjected to electrophoresis in a 15% sodium dodecyl sulphate polyacrylamide gel electrophoresis (SDS-PAGE) gel and transferred on to polyvinylidene fluoride (PVDF) membranes (Millipore, ISEQ00010). Nonspecific binding was blocked with 5% nonfat milk in Tris-buffered saline with Tween 20 (TBST) for 1 h at room temperature. Subsequently, the PVDF membranes were incubated at 4°C overnight with rabbit anti-LC3 (1:1000), anti-LC3A/B antibody (12741 Cell Signaling Technology, Danvers, Massachusetts, USA) and mouse anti-LAMP1 antibody (15665 Cell Signaling Technology, Danvers, Massachusetts, USA). Next, the membranes were washed with TBST and incubated with peroxidase-linked goat anti-rabbit IgG (1:5000, BS13278, Bioworld Technology Inc, Minneapolis, Minnesota, USA). Finally, the bound antibodies were visualized by using an the electrogenerated chemiluminescence (ECL) detection system (Vazyme Biotech, E411-04).

### Transmission electron microscopy (TEM)

For the ultrastructural analysis, epididymis specimens during hibernation period were separated and cut into small parts (1 mm^3^) and fixed in 2.5% (v/v) glutaraldehyde in 0.1 M phosphate-buffered saline (PBS;4°C, pH 7.4; overnight). Thereafter they were post-fixed with 1% (w/v) osmium tetroxide cold at 4°C temperature in the same buffer for 1 h and washed in buffer. The samples were dehydrated in increasing concentrations of ethanol and infiltrated with a propylene oxide-Araldite mixture for embedding in Araldite. The blocks were sectioned, and ultrathin sections (50 nm) were mounted on Formvar-coated grids and stained with uranyl acetate and Reynolds lead citrate for 20 min per step. The sections were analyzed by transmission electron microscopy (TEM) (Hitachi H-7650; Japan) at an accelerating voltage of 80kV.

### Statistical analysis

All the quantification was measured by the ImageJ [42] and analyzed statistically and presented by Origin Pro 2018. Region of interest (ROI) was specified at epididymal epithelium apices. For quantification analysis total 20 tissue sections were analyzed; equal number of tissue sections (n = 10) were taken from hibernation and non-hibernation group. Results were presented as mean ± SEM. The statistical significance of differences among the mean was analyzed by t-test (*P* < 0.05).
